# *Colletotrichum graminicola* keratitis: First case report from India

**DOI:** 10.4103/0301-4738.67058

**Published:** 2010

**Authors:** Prakash P Yegneswaran, Vijaya Pai, Indira Bairy, Sulatha Bhandary

**Affiliations:** Department of Microbiology, Kasturba Medical College, Manipal University, Manipal, India; 1 Department of Ophthalmology, Kasturba Medical College, Manipal University, Manipal, India

**Keywords:** Coelomycetous fungi, *Colletotrichum graminicola*, keratomycosis

## Abstract

*Colletotrichum graminicola* is a medically important fungus belonging to the order Melanconiales under the class Coelomycetes. The members of the genus *Colletotrichum* are primarily plant pathogens which cause anthracnoses (fungal infection in plants). In the past few decades, they are progressively being implicated as etiological agents of subcutaneous hyalohyphomycoses and keratomycoses. Of the five medically important members in the genus *Colletotrichum*, keratitis due to *Colletotrichum graminicola* is rare. We diagnosed *Colletotrichum graminicola* keratitis in a 44-year-old man who presented with a non-healing corneal ulcer since three weeks. Positive smears and cultures from the corneal scrapings established the causative organism as *C. graminicola*. The patient was treated with a combination of oral ketoconazole and topical fluconazole and natamycin. Infection resolved over 10 weeks and antimicrobials were stopped. We describe the clinical presentation and treatment outcome of *Colletotrichum graminicola* keratitis.

Coelomycetes are asexual fungi that produce their hyphae in specialized structures called conidiomata, which are often of two types, namely pycnidia and acervuli. Among the 11 known genera, *Colletotrichum*, *Nattrassia* and *Phoma* are medically important coelomycetous fungi encountered in clinical specimens.[1] *Colletotrichum* species are predominantly plant pathogens causing anthracnoses.[2] The acervular conidiomata covered with setae, producing elongated slimy conidia, and the presence of appressoria, are the key morphological features of the genus.[3] Five species of *Colletotrichum* have been reported to cause infections in humans, namely *C. coccoides*, *C. crassipes*, *C. dematium*, *C. gloeosporioides* and *C. graminicola*.[4] They have been implicated in causing keratomycosis, subcutaneous and systemic infections.[4,5] We herein report a patient with *Colletotrichum graminicola* keratitis which is the first from India and the second case worldwide.

## Case Report

**Figure 1:**
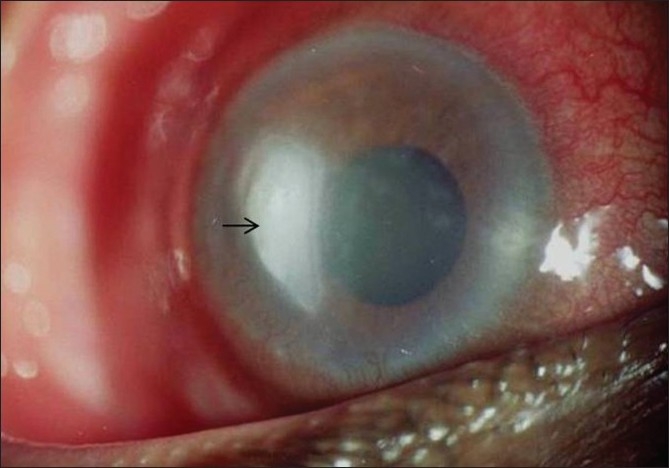
Corneal ulcer involving the temporal half of the right cornea

A 44-year-old male, a receptionist by occupation, presented to our eye department with a history of pain, redness and watering in the right eye of 25 days duration. There was no history of trauma. He had initially been seen by a local ophthalmologist, who treated him with ciprofloxacin eye drops, cycloplegics, and acyclovir ointment. His symptoms did not subside, so he was referred to our institute for further management. On presentation, the best-corrected visual acuity in his right eye was 20/500. On examination, he was found to have a corneal ulcer with infiltrate measuring 6 × 4 mm in size and epithelial defect of 6 mm involving the temporal half of the cornea [Fig. 1]. There was no hypopyon, satellite lesions or endothelial plaque. Anterior chamber showed Grade 3 cells.

**Figure 2:**
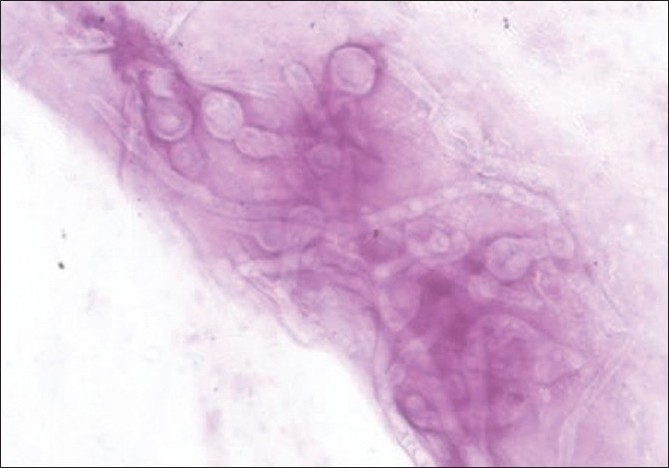
Photomicrograph of the corneal scrape specimen revealing a filamentous fungus (Giemsa Stain, ×100)

**Figure 3:**
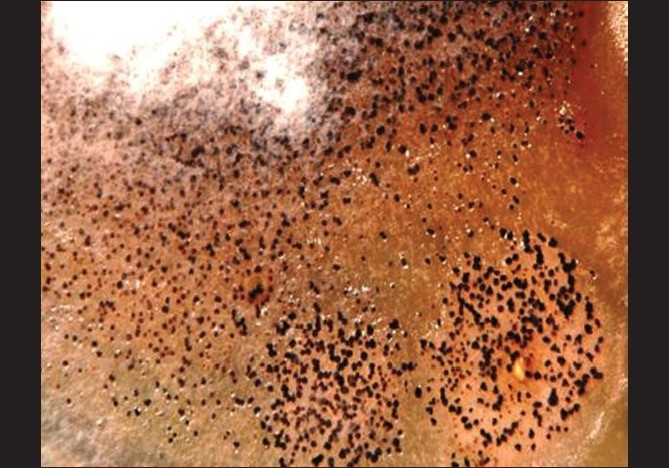
Macroscopic morphology showing salmon-colored colony with felt-like aerial mycelium. Note the numerous black sclerotia

**Figure 4:**
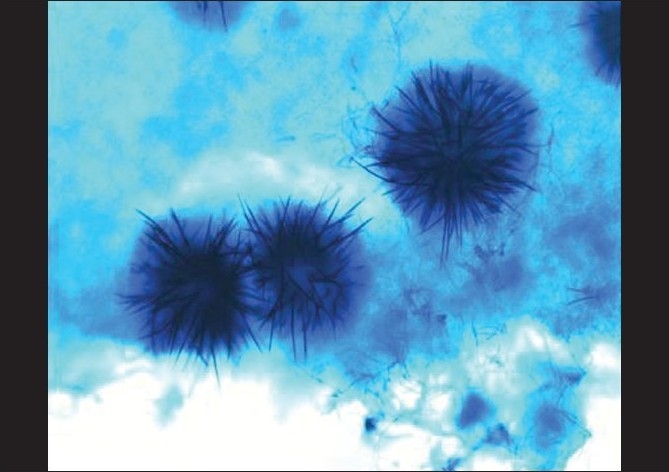
Lactophenol cotton blue tease mount preparation - 40X magnification showing abundant setae

Corneal scrapes were obtained from the active edges and smears were sent for staining with Gram, Giemsa and potassium hydroxide (KOH). Material was inoculated onto plates for bacterial, fungal and *Acanthamoeba* culture. Sabouraud’s dextrose agar (SDA) and sheep blood agar (SBA) were incubated at 28°C and 37°C, respectively. For culturing *Acanthamoeba*, non-nutrient agar with *Escherichia coli* overlay was employed and incubated at ambient temperature. Smears revealed fungal filaments [Fig. 2]. After two days, filamentous fungi was grown on SDA; gradually, at the end of two weeks, the colony assumed a salmon color with numerous black sclerotia [Fig. 3] and an orange color on the reverse that later became dark brown. A lactophenol cotton blue tease mount preparation from the colonies showed abundant setae [Fig. 4] wide, falcate, fusiform conidia gradually tapering at the apex and base and abundant appressoria with irregular margins. The fungus was initially identified as *Colletotrichum graminicola* and further confirmation carried out at the Centraalbureau voor Schimmelcultures (CBS), Fungal Biodiversity Centre, Utrecht, Netherlands. Antifungal susceptibility testing for the isolate was performed using the Clinical and Laboratory Standards Institute Document M38-A2. The minimum inhibitory concentration (expressed as microgram per milliliter) for amphotericin, itraconazole and ketaconazole at 48 h and 72 h was found to be 0.5/1, 0.5/1 and 1/2 respectively. SBA was sterile after 48 h of aerobic incubation and there was no culture recovery of *Acanthamoeba*.

**Figure 5:**
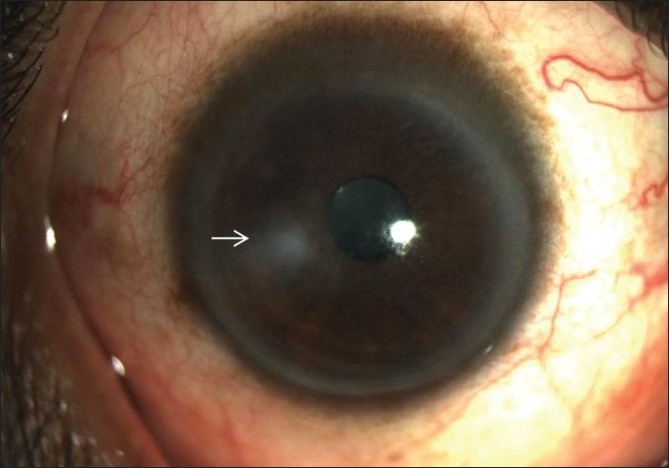
Healed ulcer with scarring at 21 weeks

Treatment was started with oral ketoconazole 200 mg twice daily (liver functions were normal) which was continued for two weeks. Fluconazole 0.3% eye drops, natamycin 5% eye drops were given hourly and atropine 1% eye drops was used thrice a day. Epithelial defect healed by six weeks and the infiltrates fully resolved by eight weeks [Fig. 5]. After 10 weeks, all medications were tapered and stopped. At 21 weeks, patient had a corneal opacity and the unaided corrected visual acuity was 20/30 and N6.

## Discussion

*Colletotrichum* is a ubiquitous fungus with a well-documented phytopathogenic potential. It is most frequently isolated from soil and plant vegetation, and has subtle morphological features making identification in culture difficult. The falcate conidia can be confused with *Fusarium* spp., a common agent of keratomycosis. Among the five medically important *Colletotrichum* species, falcate conidia are present only in *C. dematium* and *C. graminicola*. The characteristic presence of 4-6 µm-wide conidia and irregular margins of appressoria seen in *C. graminicola* helps in easy delineation of the species from *C. dematium*, which has a 3-4 µm-wide (narrower) conidia and smooth margins of appressoria.[3] SDA can be used for primary isolation of *Colletotrichum* from corneal scrapes. To enhance appresoria and sclerotia formation water agar with added plant tissue, carnation leaf agar and oatmeal agar have been recommended.[3]

In the literature, the principal *Colletotrichum* species implicated in keratomycosis has been *C. dematium* along with a documented report of *C. gleosporoides*.[6–9] In most instances, speciation of isolated *Colletotrichum* species was not attempted; hence the exact frequency of isolation of *C. graminicola* remains obscure. Besides ocular trauma, insulin-dependent diabetes mellitus and prolonged use of corticosteroids have been reported as risk factors.[4,5] Our patient, had no such risk factors.

Earlier studies have reported complete resolution of *Colletotrichum* corneal ulcers with good visual recovery following treatment with natamycin, amphotericin B with azoles, 5 flucytosine with ciprofloxacin.[5] Ritterband *et al*. first reported keratitis due to *C. graminicola* that was difficult to treat and therapeutic penetrating keratoplasty was performed twice after which there was no recurrence of fungal infection.[10] In the present case we were able to treat successfully within eight weeks by a combination of azoles and natamycin. Based on the sensitivity report a combination of amphotericin B with azole and/or natamycin with dosing regimen extending 47 ± 14 days is found to be effective in the therapeutic management of *Colletotrichum* keratitis.

Coleomycetous fungi belonging to the genus *Colletotrichum spp*. are opportunistic agents involved in keratomycoses. We report a rare case of *C. graminicola* keratitis. The report also highlights the successful treatment outcome of *C. graminicola* keratitis using a natamycin and azole combination.
